# The Use of Herbal Medicine in Sudden Sensorineural Hearing Loss in Diabetic Patients

**DOI:** 10.22038/IJORL.2023.69813.3368

**Published:** 2023-07

**Authors:** Ardavan Tajdini, Alireza Karimi yazdi, Hamideh Ravand, Leyla Sahebi

**Affiliations:** 1 *Otorhinolaryngology Research Center, Amir Alam Hospital, Tehran University of Medical Sciences, Tehran, Iran.*; 2 *Otorhinolaryngology Research Center, Imam Khomeini Hospital Complex, Tehran University of Medical Sciences, Tehran, Iran.*; 3 *Maternal, Fetal and Neonatal Research Center, Family Health Research Institute, Tehran University of Medical Sciences, Tehran, Iran.*

**Keywords:** Curcumin, Piperine, Gingerol, Sudden Sensorineural Hearing Loss, Diabetes

## Abstract

**Introduction:**

This study was conducted to evaluate the effect of Doluperine® capsule (curcumin, piperine, and gingerol) on hearing recovery in diabetic patients with Sudden Sensorineural Hearing Loss (SSNHL).

**Materials and Methods:**

Fifty-one diabetic patients with SSNHL were randomized to receive two placebo capsules (group 1), a Doluperine® plus one placebo capsule (group 2), or two Doluperine® capsules (group 3). Moreover, all patients had an injection of dexamethasone in the middle ear.

**Results:**

The proportion of significant positive changes in PTA, SDS, and SRT was 45.4%, 45.4%, and 36.37% in group1, 84.6%, 84.6%, and 76.92% in group 2, and 70%, 50.0%, and 80.0% in group 3, respectively. Many patients in group 3 did not respond to treatment in the first month, while they recovered at the end of the second month. The chance of recovery reduced with increased time between the onset of symptoms and treatment (delayed visitation) in group 1; however, this finding was not seen in groups 2 and 3.

**Conclusion:**

Doluperine® is recommended as a complementary medicine along with steroid therapy for hearing loss improvement in diabetic patients; moreover, this herbal medicine seems to play an important role in recovery in patients with delayed visitation.

## Introduction

Sudden Sensorineural Hearing Loss (SSNHL) is commonly defined as hearing loss of at least 30 dB at three consecutive frequencies within a maximum of 72 hours (1). While SSNHL is one of the most common types of hearing loss (1), its etiology is a challenging issue (2), and 85-90% of the cases are idiopathic (3). However, three causes are more important than the others, including viral infections, vascular diseases, and autoimmune processes (4-7).

The main drawback in the SSNHL topic is the methods used for treating the patients; for example, in the US, the basis of treatment is oral corticosteroids, and other therapies such as intratympanic (IT) injections are administered in later steps (8). Some studies have shown that IT injection is relatively safe and efficient (9-11). In 2016, Gao et al. conducted a meta-analysis and concluded that combination (IT and systemic) therapy was associated with advantages in the recovery rate (12). In a study conducted in Iran, a combination of IT- dexamethasone and systemic steroids increased the recovery rate in poor-prognosis SSNHL patients (13). However, oral steroid therapy is contraindicated in some patients, such as diabetic patients, pregnant women, and patients with glaucoma (14,15). 

Another important issue is the timely referral of patients for treatment, and studies have shown a strong correlation between visitation time and response to steroid therapy (16,17).

 According to the International Diabetes Federation (IDF), about 415 million adults have diabetes, increasing to 642 million in 2040 (18). There were over 5.3 million diabetic patients in Iran in 2020 (18). SSNHL is a common complication in diabetic patients (14). Due to the limitations of diabetic patients in using corticosteroids, administering natural corticosteroids as adjuncts to IT injection may be more effective than using IT injection alone. Several animal studies have documented the effect of curcumin on hearing recovery (19-24). 

Curcumin, the most active constituent of turmeric, is a natural corticosteroid with strong anti-inflammatory, antioxidant, hypoglycemic wound-healing, and antimicrobial activities and is used for preventing and treating a wide range of human diseases, especially autoimmune disorders (25). The pharmacological safety of curcumin, along with its anti-inflammatory properties, makes it suitable for medical treatment (25) 

The physiological effects of curcumin are only possible if it is combined with piperine to increase its systemic bioavailability (26). Ginger is also part of the Ayurvedic health tradition. Its main characteristics include antimicrobial, antithrombotic, anti-inflammatory, and anti-cancer properties (27).

Doluperine® includes turmeric extract highly rich in curcumin (95%), ginger extract titrated in gingerol (5%), and pepper extract (95% piperine). One capsule of Doluperine® provides 300 mg of curcumin in combination with 3.25 mg of piperine and 7.5 mg of gingerol (28). This clinical trial aimed to evaluate the impact of a combination of curcumin, piperine, and gingerol (Doluperine®) as a supplement and IT injection on SSNHL in diabetic patients.

## Materials and Methods

This triple-blind, placebo-controlled trial (IRCTID: IRCT2017012332132N1) was conducted on 51 type-2 diabetic patients with SSNHL in Amir-Alam Hospital, Tehran, Iran, which were diagnosed and referred by the otorhinolaryngologist. The patients were randomly assigned to three arms using block randomization with a block size of six. This study was approved by the Ethics Committee of Tehran University of Medical Sciences (IR.TUMS.VCR.REC.1395.1271).

Type 2 diabetic patients aged 20-65 years who had SSNHL were included in the study. The exclusion criteria were any cardiovascular or gastrointestinal disease, history of hypoglycemia, anticoagulation treatment, pregnancy, and lactation. The patients were screened by physical examination, audiometry, and complete blood count (CBC), and those whose hearing threshold was not measurable were excluded from the study too. The eligible patients were required to provide informed consent and complete a demographic questionnaire. The patients who were reluctant to participate in the study received routine treatment. Each arm was allocated using the “randomization and online databases for clinical trials” website. Patients were selected using the convenience sampling method. 

**Fig 1:**
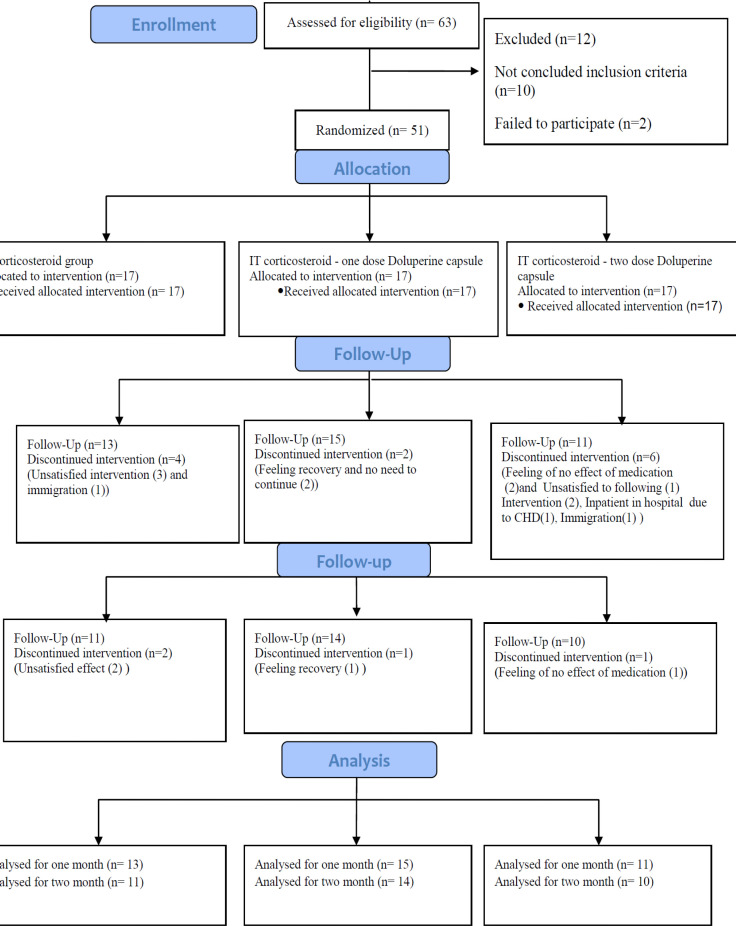
Flowchart of trial procedure

This study had three arms. The first arm included patients receiving an IT injection of dexamethasone in the middle ear and a placebo (capsules containing corn powder) twice a day for two months. The patients in the second arm received an IT injection of dexamethasone, one Doluperine® capsule per day, and one placebo capsule daily for two months. The third arm included patients receiving an IT injection of dexamethasone and two Doluperine® capsules daily for two months (Figure 1). 

The dose of IT dexamethasone (Sobhan Pharmaceutical Company) was 0.3-0.5 ml per injection for up to three weeks (two injections per week). 

Moreover, each Doluperine® capsule contained 300 mg curcumin, 3.25 mg piperine, and 7.5 mg gingerol. The injection was done in the supine position under the microscope with the head deviated 45° to the healthy side. After local anesthesia using a lidocaine 10% pump spray, an anterosuperior puncture was made in the tympanic membrane by an insulin syringe, and the solution was injected. The patients were instructed not to move 15-20 minutes after the injection to avoid ingestion or displacement. 

To ensure that the study had a triple-blind design, the physician in charge and all the study participants were blind to the content of the packages. 

Audiometry was performed three times, before the intervention, three days after the final injection (25th day), and on day 60 (end of treatment). In each audiometry measurement, the positive effect of treatment was defined as a difference of at least 15dB in the mean Pure Tone Audiometry (PTA) at three frequencies, a difference of at least 10 dB in Speech Reception Threshold (SRT), and a difference of at least 10% in the Speech Discrimination Score (SDS) between pre-treatment and post-treatment values. For a more detailed and trend assessment of the effect of the intervention on hearing improvement, four outcome subgroups were defined as subgroup 1: no recovery during the intervention(without any modification in two audiometry of twenty-five and sixty days), subgroup 2: hearing improvement in the first 25 days, but without change in hearing recovery from 25th day until the end of the study (positive change in audiometry on day 25 and remaining unchanged in audiometry on day 60), subgroup 3: no change in the first 25 days but, hearing improvement from the 25th day to the 60th day(No change between the first audiometry (before starting the treatment) and audiometry on the 25th day, but corrective changes in the audiometry on the 60th day), and subgroup 4: continuous recovery during the study (the progress of corrective changes in audiometry on the 25th day and audiometry on the 60^th^ day). The outcome subgroups definition in the study is shown in Supplementary Table 1S.

The degree of hearing loss was calculated using the mean threshold value (dB HL) based on the quartering method of 0.5, 1, 2, and 3 kHz by PTA. Hearing loss was classified as mild (26-40 dB), moderate (41-55 dB), moderately severe (56-70 dB), severe (71-90 dB), and profound (≥91 dB to 100 dB) (29)

## Results

Overall, 51 eligible patients were enrolled in this study from 2017 to 2018 and randomized to the IT corticosteroid group (group 1; 17 cases), IT corticosteroid plus one Doluperine® capsule per day (group 2; 17 cases), and IT corticosteroid plus two Doluperine® capsules per day (group 3; 17 cases). The injection of IT corticosteroid (dexamethasone) was continued until the 21st day (two injections per week) for all patients. Four, two, and six patients in groups 1, 2, and 3 were excluded from the study before the second audiometry (before completing the course of IT injection on the 25th day) due to migration or dissatisfaction with the results (Figure 1). Moreover, 2, 2, and 1 patients in groups 1, 2, and 3 were excluded before the third audiometry due to recovery or dissatisfaction with treatment. Finally, 11 patients in the first, 13 in the second, and 10 in the third groups were evaluated for two months. The patients followed for at least 25 days were analyzed as the intention-to-treat (ITT) population. 

The mean age (SD) of the patients was 55.54 (7.72), 58.63 (7.02), and 58.90 (7.34) years in groups 1, 2, and 3, respectively (F=0.195, P-value= 0.823).

Five patients (45.45%) in the first, five patients (38.46%) in the second, and three patients (33.3%) in the third group were female. There was no significant difference in age, gender, education level, hearing loss side, consanguinity, history of certain diseases or syndromes, and chronic drug use between the three groups (P-value>0.05) (Table 1). These comparisons were made in three time periods, including the beginning of the study, 25 days after the beginning, and two months after the beginning of the study (Table 1).

The first audiometry showed that hearing loss was profound in 17 (44.7%), severe in 7 (13.7%), moderately severe in 4 (7.8%), moderate in 4 (7.8%), and mild in 6 (11.8%) patients. 

**Table 1:** Comparison of some baseline and clinical characteristics of patients (frequency (%) and means (SD)) in three treatment groups)

	**Onset of study(51)**				**Twenty-five days after the onset of treatment (39)**				**Two months after the onset of treatment(35)**			
Variables	Group 1 (17)	Group 2 (17)	Group3(17)	P-value	Group 1 (13)	Group 2(15)	Group 3(11)	P-value	Group 1 (11)	Group 2 (13)	Group3 (10)	P-value
Age; mean(SD);Year	57.32 (7.75)	57.37 (6.22)	59.76 (6.99)	0. 561	56.92 (8.25)	57.53 (6.20)	58.63 (7.02)	0.823	55.54 (7.72)	58.63 (7.02)	58.90 (7.34)	0.567
Duration of onset of the disorder until the beginning of treatment; mean(SD); days	10.58 (9.98)	11.29 (11.49)	7.97 (6.8)	0. 783	11.92 (10.85)	12.86 (12.09)	9.54(7.8)	0.734	12.90 (11.33)	13.93 (12.5)	9.8 (8.21)	0.732
Gender; n (%) Female	7(41.2)	6(35.3)	9 (52.9)	0. 57	5(38.5)	5(33.3)	6(54.5)	0.54	5(45.5)	5(35.7)	5(33.3)	0.764
Education status; n (%)Under diploma (vs higher)	7(41.2)	12(70.6)	12 (70.6)	0. 128	6(46.2)	10(66.7)	9(81.8)	0.186	4(36.4)	9(64.3)	8(80.8)	0.114
Comorbidity*; n(%)Yes	10(27.6)	15(40.5)	12 (32.4)	0. 135	9(31.0)	12(41.4)	8(27.6)	0.892	8(36.8)	11(42.3)	7(26.9)	0.660
Special Drug use; n(%)Yes	7(22.6)	14(45.2)	10 (32.3)	0.06	6(26.1)	11(47.8)	6(26.1)	0.491	5(23.8)	10(47.6)	8(28.6)	0.281
Side of hearing loss; n(%) Left	5(29.4)	8(47.1)	5 (29.4)	0. 374	3(25.0)	4(53.8)	2(16.7)	0.173	2(18.2)	7(63.6)	2(18.2)	-

Group1: IT injection, Group2: IT injection + one dose Doluperine, Group3: IT injection + two doses Doluperine

*Carcinoma, mental disorders, congenital syndrome, and trauma history

As for the positive effect of treatment on the auditory threshold change using PTA (at the end of the second month), hearing improvement was seen in 45.4% of the patients (n=5) in group 1, 84.61% of the patients (n=11) in group 2, and 70% of the patients (n=7) in group 3 (P-value=0.122). 

Moreover, according to SDS, at the end of the second month, hearing improvement was seen in 45.4% of the patients in group 1 (n=5), 84.6% of the patients in group 2 (n=11), and 50.0% of the subjects in group 3 (n=5) (P-value=0.05). Comparison of SRT changes showed 36.37% of the patients in group 1 (n=4), 76.92% of the patients in group 2 (n=10), and 80.0% of the patients in group 3 (n=8) recovered by the end of the treatment period (P-value=0.05) (Table 2). Self-reported improvement was reported by 54.54% of the patients (n=6) in group 1, 92.31% of the patients in group 2 (n=12), and 80% of the patients in group 3 (n=8) (X^2^=7.32, P-value=0.026).

**Table 2:** Comparison of successful recovery in the three groups

**Intention to treat population** **( Twenty-five days after the start of treatment)**					**Final population** **(2 months after the start of treatment)**				
**Outcome**	**Group 1 (13); n (%)**	**Group2 (15); n (%)**	**Group 3 (11); n (%)**	P- Value*	**Outcome**	**Group 1 (11); n (%)**	**Group 2 (13); n (%)**	**Group3 (10); n (%)**	**P- Value ** ^*^
No recovery by PTA	7(53.85)	2(13.33)	4(36.36)	0.07	No recovery by PTA	6(54.5)	2(13.4)	3(30.0)	0.122
Recovery by PTA	6(46.15)	13(86.67)	7(63.63)		Recovery by PTA	5(45.4)	11(84.6)	7(70.0)	
No recovery by SDS	7(53.85)	3(20.0)	6(54.54)	0.108	No recovery by SDS	6(54.5)	2(15.38)	5(50.0)	0.05
Recovery by SDS	6(46.15)	12(80.0)	5(45.45)		Recovery by SDS	5(45.40)	11(84.6)	5(50.0)	
No recovery by SRT	9(69.23)	3(21.4)	2(18.18)	0.008	No recovery by SRT	7(63.63)	3(23.08)	2(20.0)	0.05
Recovery by SRT	4(30.77)	12(80.0)	9(81.81)		Recovery by SRT	4(36.37)	10(76.92)	8(80.0)	

Group1: IT injection, Group2: IT injection + one dose Doluperine, Group3: IT injection + two doses Doluperine *P-value based on chi-square

According to PTA, no patient in group 1 (0%), five patients in group 2 (38.46%), and five patients in group 3 (50%) experienced no change in the first 25 days but showed improvement from day 25 to day 60(subgroup 3(. The chi-square for linear trend showed a significant relationship between the dose of Doluperine in the study groups and hearing improvement according to PTA (group 1 was the baseline group in the exposure level) (P-Value=0.008).

According to SDS, 18.18% of the patients in group 1 (n=2), 7.69% of the patients in group 2 (n=1), and 30.0% of the patients in group 3 (n=3) experienced no change in the first 25 days but showed improvement from day 25 to day 60(being in subgroup 3((P-value by linear trend= 0.407). These figures were 9.1% in group 1 (n=1), 15.38% in group 2 (n=2), and 30% in group 3 (n=3) according to SRT (P-value by linear trend= 0.370) (Table 3). 

**Table 3:** Hearing improvement changes in three groups

**Intention to treat population**				**Final population**			
Outcome subgroups	Group1(13)	Group2(15)	Gruop3(11)	Outcome subgroups	Group1(11)	Group2(13)	Group3(10)
Subgroup1 by PTA	7(53.85)	2(13.33)	4(36.36)	Subgroup1 by PTA	6(54.5)	2(13.4)	3(30.0)
Subgroup2 by PTA	1(7.69)	2(13.33)	0(0.0)	Subgroup2 by PTA	0(0)	0(0)	0(0)
Subgroup3 by PTA	0(0.0)	5(33.33)	5(45.45)	Subgroup3 by PTA	0(0)	5(38.46)	5(50.0)
Subgroup4 by PTA	5(38.46)	6(40.01)	2(18.19)	Subgroup4 by PTA	5(45.4)	6(46.15)	2(20.0)
Subgroup1 by SDS	7(53.85)	3(20.0)	6(54.54)	Subgroup1 by SDS	6(54.54)	2(15.38)	5(50.0)
Subgroup2 by SDS	1(7.69)	1(6.67)	0(0)	Subgroup2 by SDS	1(9.1)	4(30.77)	0(0)
Subgroup3 by SDS	2(15.38)	1(6.67)	3(27.27)	Subgroup3 by SDS	2(18.18)	1(7.69)	3(30.0)
Subgroup4 by SDS	3(23.08)	10(66.66)	2(18.19)	Subgroup4 by SDS	2(18.18)	6(46.16)	2(20.0)
Subgroup1 by SRT	9(69.23)	3(21.4)	2(18.18)	Subgroup1 by SRT	7(63.63)	3(23.08)	2(20.0)
Subgroup2 by SRT	0(0)	2(13.3)	1(9.1)	Subgroup2 by SRT	0(0)	0(0)	0(0)
Subgroup3 by SRT	1(7.69)	3(21.4)	3(27.27)	Subgroup3 by SRT	1(9.1)	2(15.38)	3(30.0)
Subgroup4 by SRT	3(23.08)	7(46.67)	5(45.45)	Subgroup4 by SRT	3(27.27)	8(61.54)	5(50.0)

Group1: IT injection, Group2: IT injection + one dose Doluperine, Group3: IT injection + two doses Doluperine Subgroup1: No recovery in study duration, Subgroup 2: Improvement between day one and day 25 and without change from day 25 to day 60, Subgroup3: Without change between day one and day 25, but recovery between day 25 and day 60, subgroup4: Continued recovery from the first day to the 60th day.

The mean visitation interval between the onset of symptoms and treatment was 9.95±9.55 days. The median (IQR) visitation interval was 7 (18.0), 7 (14.5), and 7 (10.0) days in groups 1, 2, and 3, respectively. In group 1, the median visitation interval between the onset of symptoms and treatment was shorter in recovered patients than in unrecovered patients according to PTA, SRT, and SDS (7.0 vs. 18.5 days, seven vs. 11.0 days, and seven vs. 11, respectively). However, the median visitation interval differences between response and non-response groups were shorter in groups 2 and 3 than in group 1. Moreover, in group 3, the median visitation interval was longer in the patients that responded to treatment based on SRT and SDS compared to patients that did not respond to treatment (14 vs. 9 and 10 vs. 8.5 days) (Table 4).

**Table4:** Comparison of the distance between symptom onset until treatment on successful recovery on three groups

**Distance between symptom onset until treatment; day**		**Median (IQR)**	**P-Value** **
Group1	No recovery by PTA(6)	18.5(27.5)	0.589
	Recovery by PTA(5)	7.0(8.0)	
Group2	No recovery by PTA(2)	18.5(23)*	0.410
	Recovery by PTA(11)	7.0(15.0)	
Group3	No recovery by PTA(3)	10.0(12.0)	1.0
	Recovery by PTA(7)	7.0 (10.0)	
Group1	No recovery by SRT(6)	11.0(27.0)	0.788
	Recovery by SRT(5)	7(20.25)	
Group2	No recovery by SRT(2)	8.0(28)*	0.291
	Recovery by SRT(11)	7.0 (15)	
Group3	No recovery by SRT(6)	9(14)	0.400
	Recovery by SRT(4)	14(23)	
Group1	No recovery by SDS (7)	11(15.5)	1.00
	Recovery by SDS (4)	7(25)	
Group2	No recovery by SDS (3)	8 (14.0)	1.00
	Recovery by SDS (10)	7(15.5)	
Group3	No recovery by SDS(2)	8.5(10)*	0.857
	Recovery by SDS (8)	10(19.0)	

Group1: IT injection, Group2: IT injection + one dose Doluperine, Group3: IT injection + two doses Doluperine

*For groups with a sample size of 2, instead of Inter Quartile Range (IQR), the difference between the maximum and the minimum value is indicated **P-value based on Mann- Whitney test

## Discussion

The present study was the first human trial of the effect of an herbal compound containing curcumin, piperine, and gingerol on SSNHL in diabetic patients. According to previous animal studies, curcumin positively affected the prevention and treatment of hearing loss (19-24). In an experimental study by Haryuna (2015), the antioxidant effect and anti-inflammatory properties of curcumin on hearing loss and deafness were investigated in rabbits. In this study, curcumin potentially affected the prevention and treatment of oxidative damage, which was statistically significant compared to the control group (24). In another study, Scarpidis et al. found that consuming curcumin in patients undergoing cochlear implants minimized the oxidative stress caused by surgery and prevented the death of hair cells and nerve endings (21). 

Furthermore, Bucak et al. found that curcumin significantly protected the cochlear morphology and functions against paclitaxel-induced ototoxicity in rats (22). Yamaguchi et al. demonstrated that curcumin significantly reduced the effect of oxidative stress on cochlear implantation in mice (23).

An animal study found that the effect of vitamin E and curcumin on hearing improvement was clinically significant. Vitamin E alone did not affect hearing loss, while curcumin alone or combined with vitamin E had a protective effect on hearing loss (30). Fotoni (2014) and Haryuna (2016) reported that curcumin was effective in treating autoimmune intoxication resulting in hearing loss (19,20).

The present clinical trial showed important findings. First, groups 2 and 3 (IT injection + Doluperine) always had a higher response to treatment compared to group 1 (IT injection only), which was statistically significant for SDS and SRT findings. 

According to PTA, the positive response to treatment was 45.5% in the first, 84.6% in the second, and 70.0% in the third group. Moreover, a comparison of groups in response to treatment based on SRT showed that response to treatment was 36.37% in group 1, 76.22% in group 2, and 80% in group 3. These values were 45.4%, 84.6%, and 50% in groups 1, 2, and 3, according to SDS, respectively. Although the drug dose did not affect response to treatment (a slight difference in results between groups 2 and 3), the positive effect of complement therapy on response to treatment was quite clear. Moon (16) found that hearing improvement began within 14 days after early steroid combination therapy in 93.1% of the patients, and complete recovery or end of change was achieved in cumulatively 80.4% of the patients within a month after treatment.

 In the present study, the proportion of significant positive change between day 25 to 60 and, without change to the 25th day (being in subgroup3), was zero in group 1 by PTA instrument, the proportion of hearing improvement between day 25 to 60 day (being in subgroup3) according to PTA was significantly higher in group 3 compared to group 2 (50% vs. 38.46%) too. Moreover, according to SDS and SRT, hearing improvement between days 25 and 60 was 30% and 30%in group 3, while 18.18% and 9.1% in group 1, respectively.

Another important finding of this study was a correlation between timely visitation and successful treatment. The chance of recovery was reduced with increased visitation time in group 1 (IT injection), and early initiation of treatment played an essential role in response to treatment. However, delayed visitation did not affect the response to treatment in groups 2 and 3.

*Limitation*

The most important limitation of the present study was its small sample size. Clinical studies with larger sample sizes are recommended. A longer treatment period (more than two months) improves treatment effectiveness.

## Conclusion

This study was the first human trial combining the herbal supplement curcumin-piperine and gingerol with steroid therapy to treat SSNHL. This compound is recommended as complementary medicine for improving hearing loss in diabetic patients. This herbal medicine plays an important role in recovery in patients with delayed visitation.
